# Pardaxin, a Fish Antimicrobial Peptide, Exhibits Antitumor Activity toward Murine Fibrosarcoma *in Vitro* and *in Vivo*

**DOI:** 10.3390/md10081852

**Published:** 2012-08-22

**Authors:** Shu-Ping Wu, Tsui-Chin Huang, Ching-Chun Lin, Cho-Fat Hui, Cheng-Hui Lin, Jyh-Yih Chen

**Affiliations:** 1 Department of Aquaculture, National Taiwan Ocean University, Keelung 202, Taiwan; Email: wspping@gmail.com (S.-P.W.); chenghui@mail.ntou.edu.tw (C.-H.L.); 2 Marine Research Station, Institute of Cellular and Organismic Biology, Academia Sinica, 23-10 Dahuen Rd., Jiaushi, Ilan 262, Taiwan; Email: tsuichin@gmail.com; 3 Institute of Cellular and Organismic Biology, Academia Sinica, Taipei 115, Taiwan; Email: zoocclin@gate.sinica.edu.tw

**Keywords:** pardaxin, fibrosarcoma, antitumor

## Abstract

The antitumor activity of pardaxin, a fish antimicrobial peptide, has not been previously examined in *in vitro* and *in vivo* systems for treating murine fibrosarcoma. In this study, the antitumor activity of synthetic pardaxin was tested using murine MN-11 tumor cells as the study model. We show that pardaxin inhibits the proliferation of MN-11 cells and reduces colony formation in a soft agar assay. Transmission electron microscopy (TEM) showed that pardaxin altered the membrane structure similar to what a lytic peptide does, and also produced apoptotic features, such as hollow mitochondria, nuclear condensation, and disrupted cell membranes. A qRT-PCR and ELISA showed that pardaxin induced apoptosis, activated caspase-7 and interleukin (IL)-7r, and downregulated caspase-9, ATF 3, SOCS3, STAT3, cathelicidin, p65, and interferon (IFN)-γ suggesting that pardaxin induces apoptosis through the death receptor/nuclear factor (NF)-κB signaling pathway after 14 days of treatment in tumor-bearing mice. An antitumor effect was observed when pardaxin (25 mg/kg; 0.5 mg/day) was used to treat mice for 14 days, which caused significant inhibition of MN-11 cell growth in mice. Overall, these results indicate that pardaxin has the potential to be a novel therapeutic agent to treat fibrosarcomas.

## 1. Introduction

Soft-tissue sarcomas have an incidence of about two to four cases/10^5^ people and account for approximately 2.6% of sarcomas [1,2]. Treatment of soft-tissue sarcomas with doxorubicin and ifosfamide is still barely satisfactory [3]. New treatment strategies are needed, including new chemotherapeutic agents like tumor necrosis factor (TNF)-related apoptosis in esophageal, colon, pancreas, and liver carcinomas *in vitro* and *in vivo*, which was also analyzed and suggested to be an inducer of apoptosis in human fibrosarcomas [4]. To the present time, therapy of fibrosarcomas by surgical resection has been one of the choices. However, most sarcomas are located in the limbs, vessels, or nerves, which makes treatment very difficult [5,6]. Although perfusion with chemotherapeutic agents such as melphalan and TNF-α appears efficacious in some cases, melphalan and TNF-α induce side effects, and the possible loss of the treated limb is still a problem [7,8]. Therefore, establishing an available option for using antimicrobial peptides (AMPs) as a drug to cure fibrosarcomas would be a potentially valuable clinical application.

Pardaxin, a member of a family of antimicrobial peptides (AMPs) from a marine fish (*Pardachirus marmoratus*), was studied as an amphipathic polypeptide neurotoxin composed of 33 amino acids [9]. The structural features of pardaxin that are essential for its cytolytic and cytotoxic activities were defined. For example, in the lipopolysaccharide (LPS)-pardaxin 4 complex, pardaxin 4 assumes a unique helix-turn-helix conformation that looks like a “horseshoe”. The LPS-bound structure of pardaxin 4 shows noteworthy differences from structures analyzed and determined in lipid micelles and organic solvents. Solid-state nuclear magnetic resonance (NMR) experiments indicated that the membrane orientation and interactions of pardaxins were dependent on compositions of lipids in the bilayers demonstrated *C*-terminal helix and a flexible turn/helix structure at the *N*-terminal [10]. Therefore, pardaxin forms stable or transient pores in zwitterionic lipid vesicles which causes them to release their contents without loss of vesicle integrity, suggesting that pardaxin permeabilizes vesicles more efficiently by pore formation than by disruption [11]. We have previously shown that synthetic pardaxin inhibits the proliferation of HT1080 cells in a dose-dependent manner and induces programmed cell death in HeLa cells. DNA fragmentation and increases in the subG1 phase and caspase-8 activities suggested that pardaxin caused HeLa cell death by inducing apoptosis, but it had a different mechanism in HT1080 cells [12]. Pardaxin-treated HT1080 cells showed elevation of caspase-3/7 activities, disruption of the mitochondrial membrane potential, and accumulation of reactive oxygen species (ROS) production, which suggested that pardaxin may be a potential anticancer agent for selectively inducing apoptosis in cancer cells [13]. Although many AMPs such as pardaxin were identified as candidate drugs for anti-cancer treatment, there are no reports of pardaxin being used to treat tumors in a murine system.

Mutatect MN-11 tumor cells were derived from a poorly differentiated fibrosarcoma from a C57BL mouse and were grown subcutaneously in syngeneic C57BL/6 mice [14]. A histochemical examination of tumors from injected MN-11 tumor cells in mice revealed the presence of infiltrating host cells, neutrophils, macrophages, lymphocytes, and mast cells in the tumor microenvironment [15]. Neutrophils can produce superoxide, nitric oxide, and myeloperoxidase that cause an influx of neutrophils, which is associated with necrosis. These can be a source of chemotactic factors that promote the infiltration of neutrophils, which can cause chronic inflammation [15]. Therefore, appropriate activation of macrophages or immune-related gene expressions prior to surgery [16] is one method to reduce some of the detrimental effects caused by surgical operations to remove fibrosarcomas from patients. Recently, experimental results confirmed the genomic instability of cancer cells and showed a feature of tumor progression [14,15]. The contribution of AMPs to inhibiting tumor growth and reducing products of inflammatory cells has not been assessed in experimental studies. Most AMPs possess antibacterial and immunomodulatory functions against pathogen infections in hosts [17,18,19]. Treating fibrosarcoma cells with pardaxin can be employed to trigger caspase-dependent and ROS-mediated apoptosis [13]; hence, using an AMP to treat cancer patients either by inhibiting tumor growth or preventing bacterial infections may play an important role in clinical applications.

The present study was designed to further explore the mechanism of action of pardaxin therapy against fibrosarcomas. For this purpose, we established *in vitro* and *in vivo* mouse models of fibrosarcomas and examined the effect of treatment on the expressions of immune-related genes. We thus performed *in vitro* and *in vivo* analyses to demonstrate that pardaxin suppressed tumor growth and was involved in immune-related gene expression in fibrosarcoma HT-1080 cells. Our results showed that synthetic pardaxin-induced apoptosis in HT-1080 cells was caspase-dependent and ROS-mediated.

## 2. Results

### 2.1. Effects of Pardaxin on Cytotoxicity, Clonal Growth, Membrane Structure, and Cell Motility of MN-11 Cells

**Figure 1:**
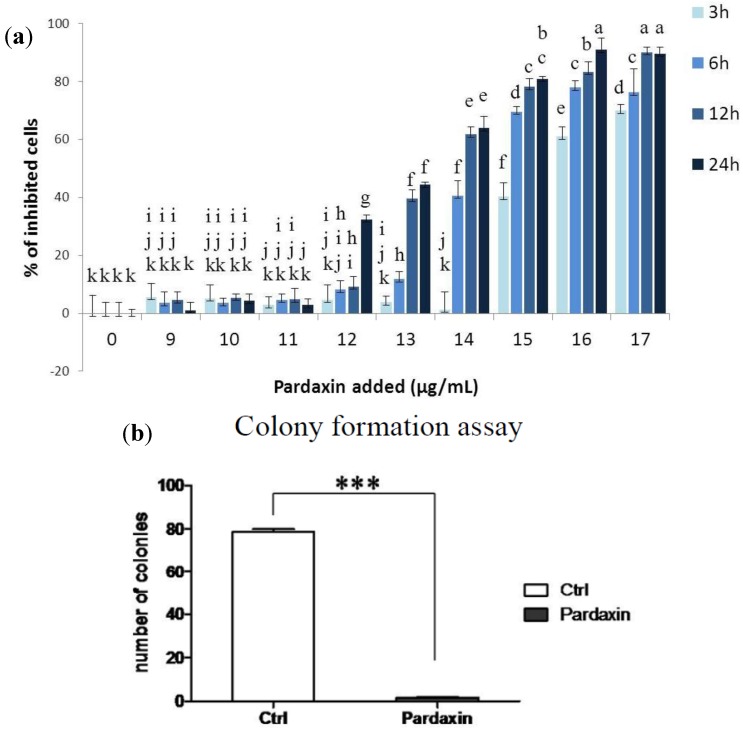
Effects of pardaxin treatment on MN-11 cells. (**a**) MN-11 cells were treated with different doses of pardaxin for 3, 6, 12, and 24 h and then monitored by an MTS assay. (**b**) Clonal assays of MN-11 cell lines treated with 13 µg/mL pardaxin or 0 µg/mL (control, Ctrl) indicated reduced colony formation. The control (Ctrl) represents no pardaxin treatment. Each bar represents the mean value of three determinations with the standard error (SE). Data with different letters significantly differ (*p* < 0.05) among treatments. Statistical analysis was performed with *t*-test to compare two groups. Multiple group comparisons were tested using analysis of variance (ANOVA) in SPSS software. Differences were defined as significant at *p* < 0.05 and <0.01. Different letters indicate a significant difference between two groups, while the same letter indicates no difference between two groups.

To understand the effects of the synthesized pardaxin on MN-11 cells, pardaxin was administered at different concentrations (0–17 µg/mL) and for different times (3, 6, 12, and 24 h), and cell viability was determined by an MTS assay (Figure 1a). Results showed higher growth inhibition ratios after 13 µg/mL treatment with pardaxin, which indicated that pardaxin was very effective at inhibiting the growth of this murine fibrosarcoma cell line. Figure 1b shows results of the clonal assay for MN-11 cells, from which it is evident that 33 amino acids (aa) of the synthesized pardaxin peptide (13 µg/mL) caused >90% inhibition of colony formation by MN-11 cells. In our previous study, we found that the synthesized pardaxin peptide caused >20% inhibition of colony formation in HT1080 cells [12]. Inhibition of colony formation in these two fibrosarcoma cell lines (HT1080 and MN-11) indicates that pardaxin exhibits a potential antineoplastic biological function. 

### 2.2. Pardaxin Induced Apoptosis of MN-11 Cells

**Figure 2:**
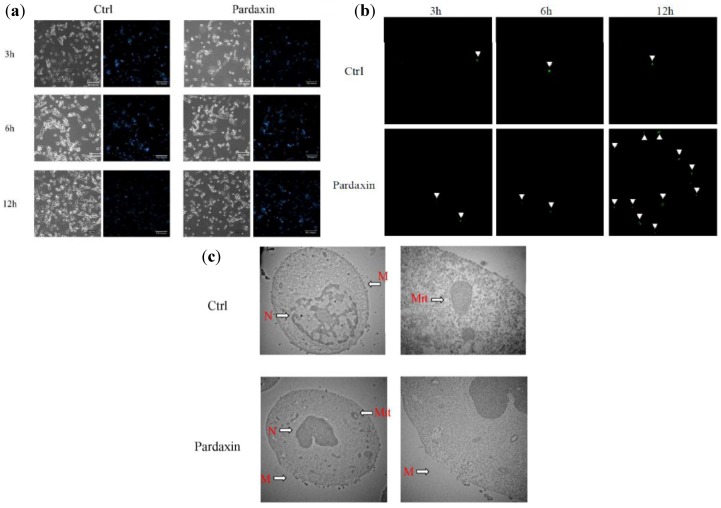
Morphological changes in MN-11 cells after exposure to different concentrations (0 and 13 µg/mL) of pardaxin for 3, 6, and 12 h. (**a**) Detection of typical features of apoptotic nuclear condensation by Hoechst 33258 staining (magnification 200×). (**b**) Caspase-3/7 activity was measured after 3, 6, and 12 h of treatment, and green color was detected under a fluorescence microscope. The green color is indicated by white arrowheads. (**c**) Effects of pardaxin on membranes of MN-11 tumor cells examined by transmission electron microscopy. Untreated cells (Ctrl) showed a normal surface, while cells treated with pardaxin (13 µg/mL) for 24 h revealed disrupted cell membranes. N, nuclear; M, cell membrane; Mit, mitochondria.

Hoechst 33258 staining was used to investigate changes in cell nuclei (Figure 2a). MN-11 cells treated with pardaxin (13 μg/mL) showed apoptotic bodies containing nuclear fragments, and the chromatin had become condensed or marginalized. The nuclear envelope appeared lytic, and the cytoplasm was shrunken. Some of these same features appeared in untreated cells due to MN-11 cells being very sensitive to mutations by clastogenic agents [14]. When treated with pardaxin (13 μg/mL) for 3, 6, and 12 h, greater numbers of apoptotic cells were observed (Figure 2a). Furthermore, to determine if pardaxin-induced apoptosis occurred in a time-dependent manner, MN-11 cells were incubated with pardaxin (13 μg/mL) for 3, 6, and 12 h and then analyzed with a caspase-3/7 green detection reagent. Pardaxin treatment (13 μg/mL) for 3, 6, and 12 h produced increased green color compared to the untreated group, suggesting that pardaxin increased the apoptotic rate in a time-dependent manner (Figure 2b). Apoptosis induced by pardaxin was analyzed by examining membrane disruption and mitochondrial damage by TEM. When treated with pardaxin, mitochondria appeared hollow, nuclei were condensed, and cell membranes were disrupted (Figure 2c). Thus, apoptosis and lytic characters co-exist and are the potential mechanisms of pardaxin-induced cell death.

### 2.3. Pardaxin Inhibits Tumor Growth and Vascularization *in Vivo*

**Figure 3:**
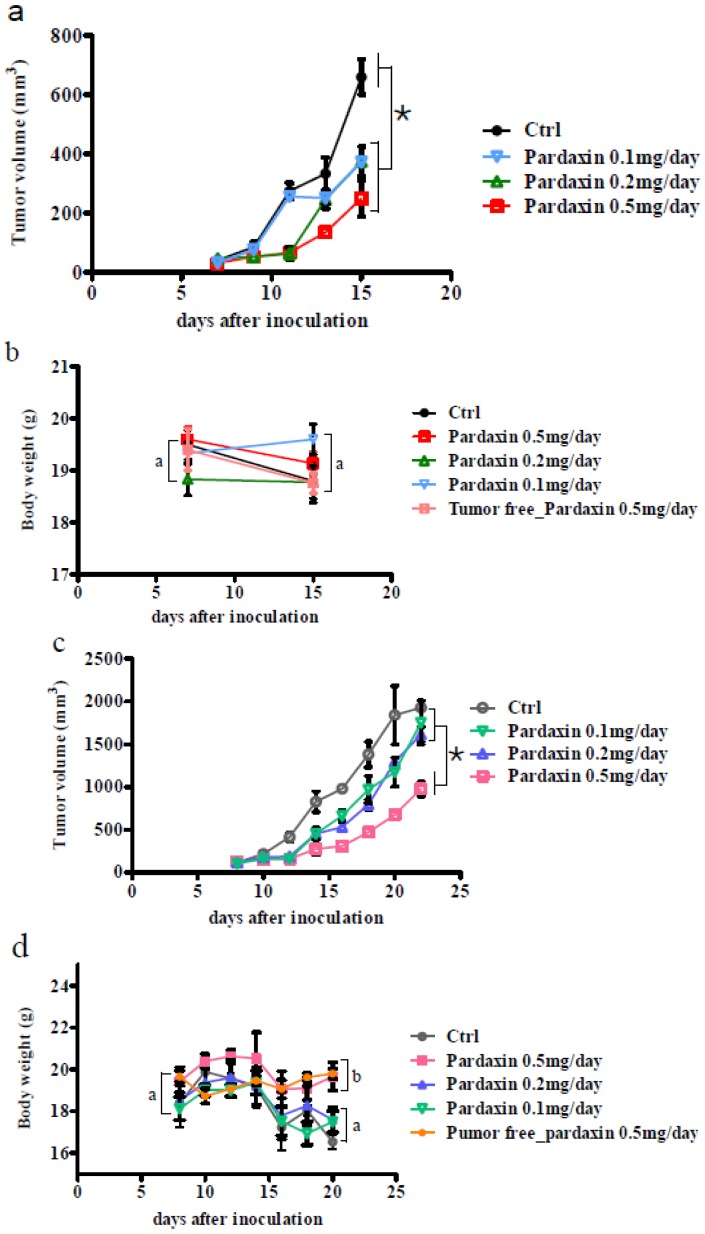
Pardaxin inhibited tumor growth *in vivo*. (**a**) The first day of pardaxin administration was labeled day seven. Pardaxin was injected for 7 days of treatment. Control mice were injected with only PBS. Each mouse was injected with a different concentration of pardaxin, and results are plotted using different colors. Individual tumor sizes are shown as the tumor volume. (**b**) The body weight was recorded from the first day of pardaxin administration and labeled days 7–15. Pardaxin appeared to be nontoxic to normal cells. When administered at 500 µg/day, which inhibited tumor growth, there were no notable side effects on the body weight compared to mice with no tumor growth at the end of the experiment. (**c**) The first day of pardaxin administration was labeled day seven. In total, pardaxin was injected for 14 days of treatment. Control mice were injected with only PBS. Mice were injected with different concentrations of pardaxin, and these are plotted in different colors. Individual tumor sizes are shown as the tumor volume. (**d**) The body weight was recorded from the first day of pardaxin administration and labeled days 7–21. Pardaxin appeared to be nontoxic to normal cells. When administered at 500 µg/day, which inhibited tumor growth, there were no notable side effects on the body weight of mice compared to those with no tumor growth at the end of the experiment. The tumor region had decreased compared to the control group. Differences were defined as significant at *p* < 0.05. Different letters (a, b, c, d, and e) or an asterisk (*) indicate a significant difference between two groups, while the same letter indicates no difference between two groups.

**Figure 4:**
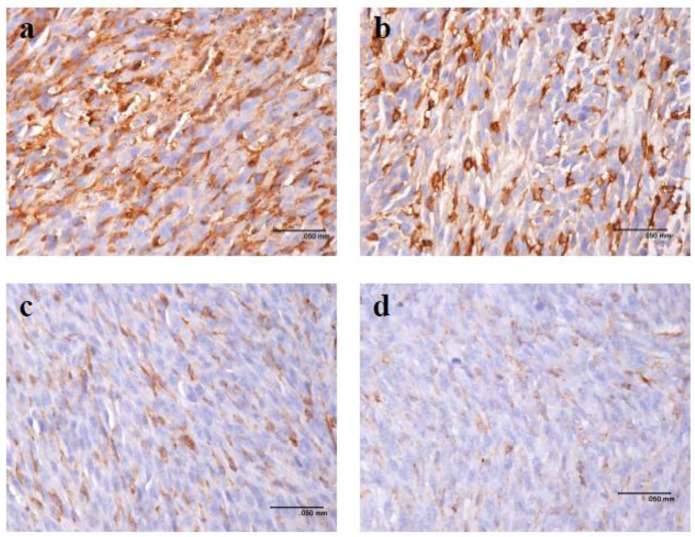
Pardaxin inhibited vascularization *in vivo*. Vessels in tumors were stained with an anti-CD31 antibody. CD31-positive vessels decreased from a to d. (**a**) Mice not treated with pardaxin; and those injected with a (**b**) low dose (5 mg/kg; 0.1 mg/day), (**c**) medium dose (10 mg/kg; 0.2 mg/day), and (**d**) high dose (25 mg/kg; 0.5 mg/day).

To investigate whether pardaxin suppresses tumor progression *in vivo*, MN-11 cells were subcutaneously implanted into C57BL/6 mice to generate a tumor xenograft model. Seven days post-implantation, all animals had developed palpable tumors. At the end of the experiments, the tumor growth rate was substantially lower in mice given cells injected with a high dose (25 mg/kg; 0.5 mg/day) of pardaxin compared to the control group as judged by the tumor volume (Figure 3a,c). Following 7 (Figure 3a) or 14 days (Figure 3c) of pardaxin treatment, the average tumor volume of the pardaxin group injected with a high dose (25 mg/kg; 0.5 mg/day) was much smaller than those of the pardaxin group injected with a low dose (5 mg/kg; 0.1 mg/day) and the control group. It should be noted that body weights of animals injected with pardaxin at a high dose (25 mg/kg; 0.5 mg/day), medium dose (10 mg/kg; 0.2 mg/day), and low dose (5 mg/kg; 0.1 mg/day) and treated for either 7 (Figure 3b) or 14 days (Figure 3d) did not significantly differ. Pardaxin appeared to be nontoxic to normal cells, and when administered at a high dose (25 mg/kg; 0.5 mg/day), it inhibited tumor growth, but there were no notable side effects on the body weight of mice (the mice did contain tumors) at the end of the experiment. When tumor sections were immunohistochemically stained with an anti-CD31 antibody, the expression of CD31 in the tumor was reduced in mice injected with high-dose pardaxin (25 mg/kg; 0.5 mg/day) for 14 days (Figure 4d), whereas the expression of CD31 of the control (untreated) group remained at a relatively high level (Figure 4a). Following increases in the pardaxin dose, anti-CD31 antibody staining gradually decreased (Figure 4). The data suggest that pardaxin retarded tumor development by suppressing both cell proliferation and tumor vascularization.

### 2.4. Gene Expression after Treatment with Pardaxin in Tumor-Bearing Mice

**Figure 5:**
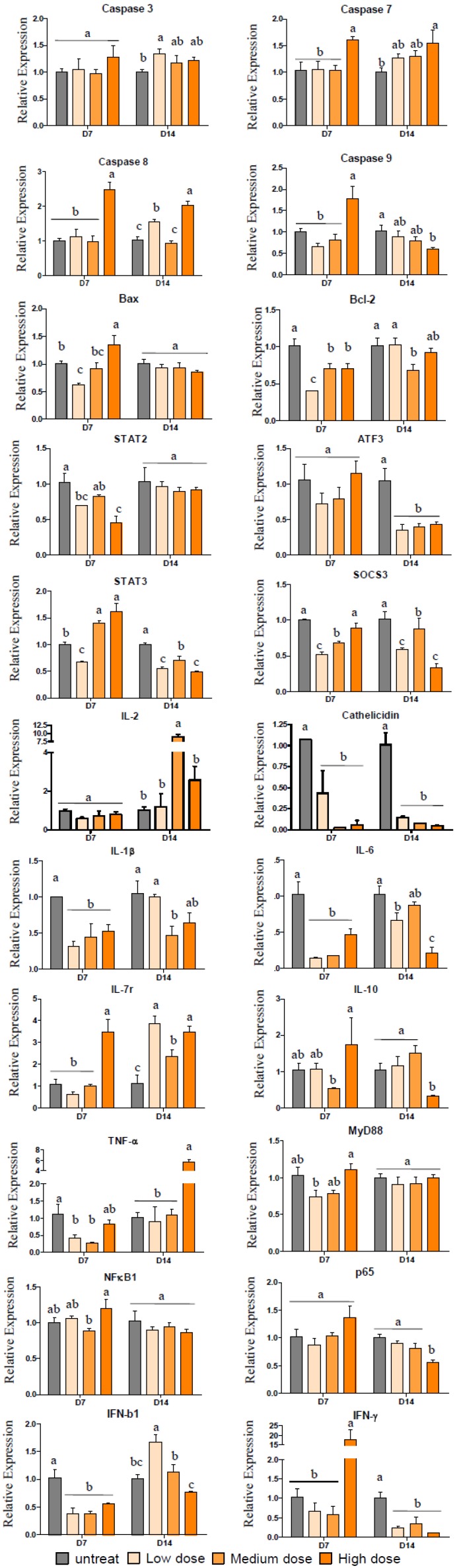
Quantification of transcript levels by a comparative real-time RT-PCR. RNA from tumor masses from mice untreated (control), or treated with a low dose (5 mg/kg; 0.1 mg/day), medium dose (10 mg/kg; 0.2 mg/day), and high dose (25 mg/kg; 0.5 mg/day) of pardaxin for 7 or 14 days. The group not treated with the peptide served as the control, “untreated” for the gray box. Samples were collected after treatment for 7 or 14 days. The transcript abundance, normalized to actin expression, is expressed as the relative expression and graphed on a rational scale. Each bar represents the mean value from three determinations with the standard error (SE). Data (mean ± SE) with different letters significantly differ (*p* < 0.05) between treatments. Statistical analysis was performed with a *t*-test to compare two groups. Multiple group comparisons were tested using analysis of variance (ANOVA) in SPSS software. Differences were defined as significant at *p* < 0.05 and <0.01. Different letters indicate a significant difference between two groups, while the same letter indicates no difference between two groups.

To study the effects of pardaxin on tumor functions, we performed a real-time RT-PCR analysis of caspase-3, caspase-7, caspase-8, caspase-9, Bax, Bcl-2, STAT2, ATF3, STAT3, SOCS3, IL-2, cathelicidin, TNF-α, MyD88, NF-κB1, p65, IFN-β1, IFN-γ, IL-1β, IL-6, IL-7r, and IL-10. As shown in Figure 5, we observed a tendency for decreases in caspase-9, ATF3, SOCS3, STAT3, cathelicidin, p65, and IFN-γ in MN-11 cells growing in C57BL/6 mice after treatment with both low and high doses of pardaxin for 14 days (Figure 5). We observed a tendency for increases in caspase-7 and IL-7r in MN-11 cells growing in C57BL/6 mice after treatment with both low and high doses of pardaxin for 14 days (Figure 5). On the one hand, we observed a tendency for decreases in Bcl-2, STAT2, cathelicidin, and IFN-β1 in MN-11 cells growing in C57BL/6 mice after treatment with both low and high doses of pardaxin for 7 days (Figure 5); on the other hand, we observed a tendency for an increase in STAT3 in MN-11 cells growing in C57BL/6 mice after treatment with both low and high doses of pardaxin for 7 days (Figure 5). These results suggest that pardaxin downregulated STAT2 (or STAT3), which decreased IFN-γ expression, thus inhibiting expressions of cathelicidin and cytokine (such as IL-12) genes, and may have had pleiotropic effects on MN-11 cells.

### 2.5. Cytokine Secretion in Serum of Tumor-Bearing Mice after Treatment with Pardaxin

**Figure 6:**
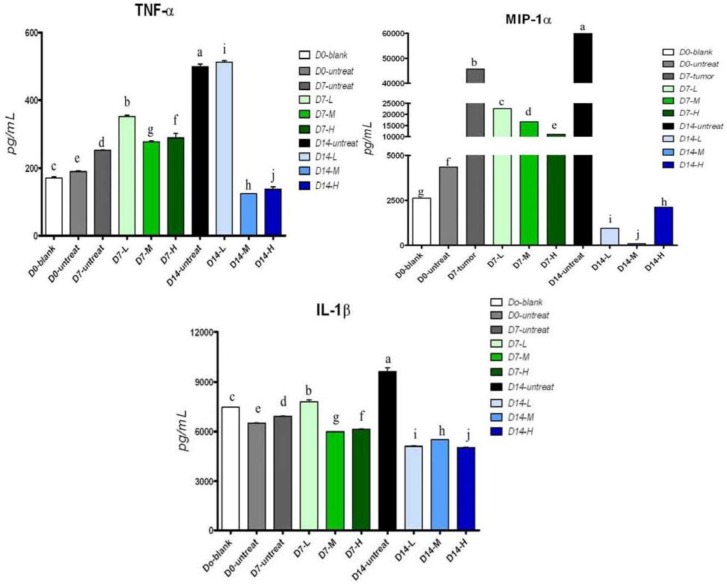
Effects of pardaxin on serum cytokine levels. Mice were injected with pardaxin at a low (L, 5 mg/kg; 0.1 mg/day), medium (M, 10 mg/kg; 0.2 mg/day), and high dose (H, 25 mg/kg; 0.5 mg/day), or left untreated (untreated) for 7 (D7) or 14 days (D14). The D0-blank was not treated with MN-11 cells or pardaxin. The D0-untreated group was injected with MN-11 cells, but received no pardaxin. Each bar represents the mean value from three determinations, with the standard error (SE). Data (mean ± SE) with different letters significantly differ (*p* < 0.05) among treatments.

To investigate the effects of pardaxin on MN-11 cell functions, we detected TNF-α, IL-1β, and MIP-1α concentrations in serum after treatment of tumor-bearing mice with pardaxin. As shown in Figure 6, results showed that TNF-α and IL-1β decreased after 14 days of treatment with both the high (25 mg/kg; 0.5 mg/day) and medium doses (10 mg/kg; 0.2 mg/day) of pardaxin compared to the untreated group. MIP-1α significantly decreased after 14 days of treatment with high (25 mg/kg; 0.5 mg/day), medium (10 mg/kg; 0.2 mg/day), and low doses (5 mg/kg; 0.1 mg/day) of pardaxin compared to the untreated group.

## 3. Discussion

Pardaxin was first isolated from *P. marmoratus* in the early 1980s, and its biological properties were initially noted: Inducing transcription of a vesicular stomatitis virus in the absence of nonionic detergents and making the virion membrane permeable to nucleoside triphosphates [20,21]. Subsequently, pardaxin became of significant interest as a potential biophysical model to study ionic channel selectivity [22] and as a potential drug for new target sites in bacteria [23]. In addition, it also showed signs of antitumor activity in *in vitro* studies with several cancer cell lines [13].

Several studies have suggested that pardaxin possesses potent antitumor activity in cancer cell lines [12,13], although the effects *in vivo* and the mechanisms behind this activity are still not very clear. MN-11 cells were derived from an MC1A fibrosarcoma in a male C57BL/10 mouse. They are very sensitive to mutations by clastogenic agents *in vitro* and *in vivo* [14]. Recent studies have suggested that pardaxin induces apoptosis by triggering caspase-dependent and ROS-mediated apoptosis in human fibrosarcoma HT-1080 cells [13]. In the present study, pardaxin caused striking concentration- and time-dependent growth inhibition of MN-11 cells. A 24-h exposure to 13 µg/mL resulted in a great inhibition ratio. Pardaxin also presented apoptosis-inducing activity. Pardaxin treatment of MN-11 cells resulted in morphological changes typical of apoptosis; these changes included condensed chromatin, nuclear envelope lysis, and shrunken cytoplasm. A colony formation assay demonstrated that pardaxin inhibited MN-11 invasion. In previous studies, pardaxin disrupted the lipid bilayer dependent on membrane components. Those results demonstrated that pardaxin was located on the surface of lipid bilayers constructed of POPC and was inserted into lipid bilayers devised of DMPC [24]. Moreover, plasma membranes of cancer cells are composed of anionic PS and O-glycosylated proteins [25,26]. Comparison of these features suggested that AMPs could not electrostatically interact with non-transformed cells such as tumor cells, the plasma membranes of which are composed of zwitterionic membrane components, such as sphingomyelin, phosphatidylethanolamine [27], and phosphatidylcholine. It was established that possessing the lipid composition allows AMPs to selectively kill malignant cells. This may be one of the mechanisms for the inhibition of tumor cell proliferation. Recently biophysical studies on pardaxin showed that pardaxins physically interact with cell membranes by forming pores or voltage-gated ion channels that disrupt cellular functions. It was shown that the membrane composition plays a vital role in its antimicrobial activity [28]. Cholesterol plays a very important role in the antitumor activities of a peptide. The addition of cholesterol and pardaxin to dimyristoyl phosphatidylcholine resulted in a very marked lowering of the transition temperature, which suggested that the peptide promoted redistribution of cholesterol in the membrane [29]. Moreover, the presence of cholesterol significantly reduced the backbone motion and the tilt angle of the *C*-terminal amphipathic helix of pardaxin, suggesting the involvement of cholesterol in the selectivity of the broad-spectrum antimicrobial activities of pardaxin [30].

At the moment, all methods of cancer treatment, including chemotherapy, radiotherapy, suicide gene therapy, immunotherapy, and surgery, strongly activate certain signal pathways such as linking to apoptosis to kill tumor cells. On the other hand, there is an urgent need for more effective and less toxic therapies for solid malignancies. Chemotherapy alone shows high toxicity and a low survival rate that may be due to cancer cells developing drug resistance [31,32]. In this study, to overcome such shortcomings, pardaxin was used as a drug for injection into the intratumoral space to eradicate surviving clonogenic cells, and showed significant results in preventing tumor growth. Pardaxin injected into tumor sites of MN-11 cells in mice also did not induce any side-effects according to our results (Supplementary Figure S1). Recently, cisplatin rapidly changed from the interstitial space to the tumor site, which prevented potentially fatal toxicities in a model of feline solid tumors with electrochemotherapy (ECT) treatment [33,34]. The ECT concept was established to increase susceptibility of tumor cells to permeabilizing pulses compared to normal tissues, and it produced semi-selective drug delivery [35,36,37], thereby diminishing side effects. Pardaxin is a membrane-lysing peptide, which indicates that a majority of host-defense peptides exert their action by permeabilizing microbial or tumor membranes [38]. Conformational analysis of pardaxin peptides and a study of their model membrane-permeabilizing activities indicated that their selective activities could be explained by their biophysical properties [39]. As described above, our research results suggested that pardaxin might be able to replace the ECT method when pardaxin is directly added to the surgical protocol. There was unanimous support of results obtained in a previous study by our group with another AMP as a drug directly injected into tumor regions which reduced tumor growth in a mouse system [40]. Indeed, one of the aims of our study was to evaluate whether AMPs (such as pardaxin) can be a future anticancer protocol through injection into tumor sites. The simple strategy of using pardaxin provides another choice for cancer therapy, while avoiding harmful or fatal side effects. It is very important to develop compounds such as AMPs that can be used to selectively kill tumor cells [41]. There are other AMPs that have been shown to have antimicrobial activities such as LL-7–27, a 21-residue peptide segment (LL-7–27; RKSKEKIGKEFKRIVQRIKDF), has been shown to exhibit potent activity against microbes but not against erythrocytes. Another 15-residue peptide named G15 was shown to exert potent activity against microbes but not against human Jurkat cells. These results show the susceptibility of bacteria and the resistance of erythrocytes and human cells and may have implications for designing membrane-selective therapeutic agents [42,43].

**Figure 7:**
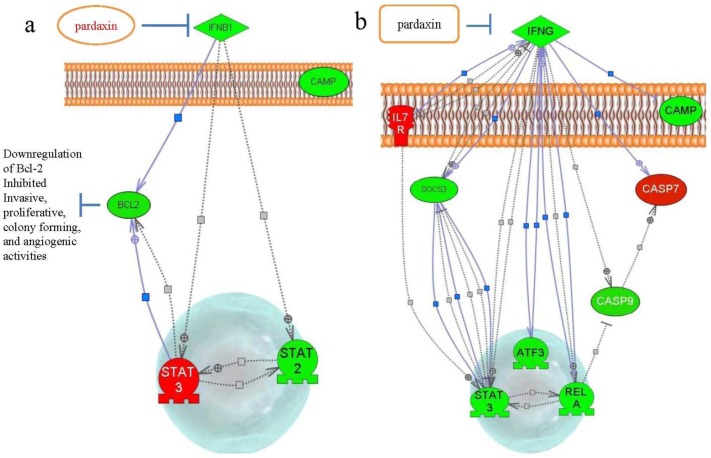
Schematic representation of the signaling cascade of pardaxin-induced apoptosis-related gene expressions after (**a**) 7 and (**b**) 14 days of treatment.

In view of emerging data that pardaxin treatment caused increased ROS production, followed by the triggering of mitochondrion-dependent apoptosis by activating caspase-3/7 activities [13], our results showed that treatment with pardaxin for 7 days downregulated IFN-β1 and Bcl-2 (Figure 5 and Figure 7a). Bcl-2 family proteins are essential regulators of mitochondrion-related apoptosis via modulation of mitochondrial membrane permeabilization to control the release of apoptosis inducers such as apoptosis-inducing factor (AIF) and cytochrome [44,45]. Downregulation of Bcl-2 gene expression may be the reason why pardaxin inhibited the invasive and proliferative properties of MN-11 cells and inhibited colony-forming and angiogenic activities [46]. Intratumoral administration of pardaxin inhibited tumor growth, possibly by downregulating Th1-type cytokine production (TNF-α and IFN-γ) and inducing apoptosis of fibrosarcomas by decreasing the Bax/Bcl-2 ratio and activating caspase-3. In this study, after treatment with pardaxin for 14 days, we found that downregulation of SOCS-3 gene expression may have led to increased death of MN-11 cells in mice (Figure 5 and Figure 7b). We found a considerable increase in activation of the proapoptotic caspase-7. We showed that pardaxin could inhibit IFN-γ and downregulate SOCS3 expression.

## 4. Experimental Section

### 4.1. Cells and Cell Culture

Fibrosarcoma MN-11 cells (murine fibrosarcoma, ATCC no.: CRL-2800; American Type Culture Collection, Manassas, VA, USA) were maintained in Dulbecco’s modified Eagle medium (DMEM, Gibco, Grand Island, NY, USA) containing 1.5 g sodium bicarbonate (Sigma, St. Louis, MO, USA) and 10% fetal bovine serum (FBS, Gibco). MN-11 cells were grown in ATCC-suggested medium. All experiments were performed in six- or 96-well culture plates dependent on the experiment.

### 4.2. *In Vitro* Cytotoxicity Assay and Soft-Agar Colony Formation Assay

Cells were cultured in complete medium in 96-well plates in this test. The number of MN-11 cells for the *in vitro* assay was 3000 cells/well. After 24 h of incubation, cells were treated with pardaxin (0–20 µg/mL) and incubated for 3, 6, 12, or 24 h. Pardaxin was synthesized and purified by GL Biochemistry (Shanghai, China) to a purity grade of >95% [12,47]. Synthetic peptides were reconstituted in phosphate-buffered saline (PBS; pH 7.4) for the experiments. Cell viability was measured at the end of treatment; 20 μL of the tetrazolium compound, 3-(4,5-dimethylthiazol-2-yl)-5-(3-carboxymethoxyphenyl)-2-(4-sulfophenyl)-2*H*-tetrazolium, inner salt (MTS) and an electron-coupling reagent (phenazine methosulfate, PMS) mixture (Promega, Mannheim, Germany) were added for 2–4 h at 37 °C. The optical density was spectrophotometrically measured at 490 nm on a microtiter plate reader. Experiments were repeated three times. Results are expressed as a percentage of the inhibition rate for viable cells, and values were deducted from the PBS-treated group. The soft-agar colony formation assay used 8000 MN-11 cells that were suspended in 2 mL of a 0.7% agar solution containing cell culture medium (2× DMEM) and 20% FBS at a final concentration of 13 μg/mL pardaxin, and layered on top of a 0.5% agar layer in six-well plates. PBS was used as the control group without adding pardaxin to the agar. Plates were incubated for 21 days at 37 °C in a humidified atmosphere containing 5% CO_2_. Cell colonies were visualized following treatment with 2 mL Giemsa stain (ACCUSTAIN, Giemsa stain, Sigma) for 60 min, destained with deionized water, and observed by light microscopy. These experiments were repeated three times, and at least five wells were replicated each time for each condition.

### 4.3. Detection of Cell Viability by Acridine Orange (AO)/Ethidium Bromide (EtBr) Staining and Transmission Electron Microscopy (TEM)

MN-11 cells (10^5^ cells/well/2 mL culture medium) were seeded in six-well plates, treated with 13 µg/mL of the synthesized pardaxin peptide for 3, 6, and 12 h, and processed for staining using cold PBS containing 1 mg/mL EtBr and 1 mg/mL AO. Cells were washed with cold PBS to remove excess dye, subsequently observed, and photographed under a fluorescence microscope. Experiments were performed in triplicate. Samples were analyzed and prepared according to a published report [42,48] and then analyzed by TEM.

### 4.4. Hoechst 33258 Assay and Caspase-3/7 Activity for Apoptosis

MN-11 cells (10^5^ cells/well/2 mL culture medium) were detected by Hoechst 33258 staining following the manufacturer’s protocol (Sigma). Briefly, cells (10^5^ cells/well/2 mL culture medium) were seeded in six-well plates for 16 h, and pardaxin was added (13 µg/mL) for 3, 6, and 12 h. The cell medium suspension was washed with PBS twice. PBS (1 mL) containing 3.7% paraformaldehyde (Sigma) was added for 150 min, and then the paraformaldehyde was removed, and cells were washed with PBS twice. Hoechst 33258 reagent (1 μg/mL) was added to stain cells in the dark at room temperature for 15 min. Stained cells were examined and immediately photographed under a fluorescence microscope (Olympus, Tokyo, Japan) at excitation wavelengths of 330–380 nm. Apoptotic cells were identified on the basis of morphologic changes in their nuclear assembly by observing chromatin condensation and fragment staining with Hoechst 33258. To measure caspase-3/7 activity, the CellEvent™ Caspase-3/7 green detection reagent was used (Invitrogen, Carlsbad, CA, USA). Briefly, subconfluent MN-11 cells (10^5^ cells/well/2 mL culture medium) in six-well plates were treated with pardaxin (13 µg/mL) for 3, 6, and 12 h. Caspase-3/7 activity was measured 3, 6, and 12 h after treatment by detection under a fluorescence microscope.

### 4.5. *In Vivo* Antitumor Efficacy of Pardaxin in Tumor Xenografts

C57BL/6 female mice (35 days old) were bought from Biolasco Taiwan (Taipei, Taiwan) and were housed under specific pathogen-free conditions. A single-cell suspension of 5 × 105MN-11 cells in 0.1 mL of PBS was injected subcutaneously into the left flank leg for 7 days, after which tumors measured about 100 mm^3^; then mice were arbitrarily separated into several test groups. Each group was composed of five C57BL/6 female mice. The trial groups were divided into a control group (untreated), and groups injected with 5 (low dose), 10 (medium dose), or 25 mg/kg (high dose) pardaxin. There were two treatment methods for the injection period: One consisted of 14 days of pardaxin injections, while the other consisted of only 7 days of pardaxin injections. The tumor size was calculated followed a previously published formula [49]. After the 7th or 14th day of injecting pardaxin into the tumors, blood from the retro-orbital plexus was collected from each mouse. The blood was sent to the Taiwan Mouse Clinic, National Comprehensive Mouse Phenotyping and Drug Testing Center [50] for analysis of glutamic oxaloacetic transaminase (GOT), glutamic pyruvic transaminase (GPT), blood urea nitrogen (BUN), creatinine (Cre), uric acid (UA), total cholesterol (TCHO), triglyceride (TG), total bilirubin (TBIL), total protein (TP), and albumin (ALB). Ethical approval was obtained from the Animal Care and Ethics Committee, NTOU.

### 4.6. Detection of Cytokine Expression Levels and Immunohistochemistry

To understand cytokine variations after injecting pardaxin to treat tumor-bearing mice or pardaxin alone in mice, we measured the cytokines, IL-1β, TNF-α, and macrophage inflammatory protein (MIP)-1α, following methods of an enzyme-linked immunosorbent assay (ELISA) development kit (Pepro Tech, NJ, USA). A murine monoclonal antibody (mAb) against the endothelial cell marker CD31 (PECAM-1, CD31, clone: MEC13.3, Santa Cruz Biotechnology, Santa Cruz, CA, USA) was used to stain biopsy specimens in tumors, which had been treated with pardaxin. Briefly, the tumor and spleen were removed, fixed in a 10% buffered neutral formalin, and processed by embedding in paraffin. Immunohistochemistry and hematoxylin and eosin staining were carried out by the Taipei Institute of Pathology [40].

### 4.7. RNA Isolation and Reverse-Transcription Qualitative Polymerase Chain Reaction (RT-qPCR)

Total RNA was isolated using Trizol (Ambion, Invitrogen) in tumors from tumor-bearing mice injected with pardaxin or tumors from mice not injected with pardaxin. To quantify immune-related gene messenger (m)RNA levels, RT reactions were carried out with 200 ng total RNA using M-MLV reverse transcriptase (ReverTra Ace qPCR RT kit, Toyobo, Tokyo, Japan). Primers for immune-related gene amplification are listed in Table 1. The RT-qPCR conditions were similar to those of our previous publication [51]. Immune-related gene expression levels were normalized to the β-actin mRNA level. Data were analyzed using the delta-delta Ct method.

**Table 1:** Primers for immune-related gene amplification.

	gene	primer (5′→3′)	product (bp)
1	mActin F′	5′-TTCGTTGCCGGTCCACACCC-3′	90
	mActin R′	5′-GCTTTGCACATGCCGGAGCC-3′	
2	mCamp F′	5′-GCCGCTGATTCTTTTGACAT-3′	108
	mCamp R′	5′-AATCTTCTCCCCACCTTTGC-3′	
3	mIL-1β F′	5′-TGTAATGAAAGACGGCACACC-3′	68
	mIL-1β R′	5′-TCTTCTTTGGGTATTGCTTGG-3′	
4	mIL-2 F′	5′-TCTGAGGAGATGGATAGC-3′	78
	mIL-2 R′	5′-TGTTGTAAGCAGGAGGTA-3′	
5	mIL-4 F′	5′-CATCGGCATTTTGAACGAG-3′	104
	mIL-4 R′	5′-CGAGCTCACTCTCTGTGGTG-3′	
6	mIL-6 F′	5′-GCTACCAAACTGGATATAATCAGGA-3′	85
	mIL-6 R′	5′-CCAGGTAGCTATGGTACTCCAGAA-3′	
7	mIL-7r F′	5′-CGAAACTCCAGAACCCAAGA-3′	61
	mIL-7r R′	5′-AATGGTGACACTTGGCAAGAC-3′	
8	mIL-10 F′	5′-CAGAGCCACATGCTCCTAGA-3′	78
	mIL-10 R′	5′-GTCCAGCTGGTCCTTTGTTT-3′	
9	mSTAT2 F′	5′-CCTGGTAAGATCCCTTTCTGG-3′	70
	mSTAT2 R′	5′-GATCCTTCAGGTGGTCGTGT-3′	
10	mSTAT3 F′	5′-GGAAATAACGGTGAAGGTGCT-3′	66
	mSTAT3 R′	5′-GGAAATAACGGTGAAGGTGCT-3′	
11	mSOCS3 F′	5′-ATTTCGCTTCGGGACTAGC-3′	126
	mSOCS3 R′	5′-AACTTGCTGTGGGTGACCAT-3′	
12	mTnfα F′	5′-TCTTCTCATTCCTGCTTGTGG-3′	128
	mTnfα R′	5′-GGTCTGGGCCATAGAACTGA-3′	
13	mMyD88 F′	5′-TGGCCTTGTTAGACCGTGA-3′	73
	mMyD88 R′	5′-AAGTATTTCTGGCAGTCCTCCTC-3′	
14	mNfkb1 F′	5′-CACTGCTCAGGTCCACTGTC-3′	78
	mNfkb1 R′	5′-CTGTCACTATCCCGGAGTTCA-3′	
15	mp65 F′	5′-CCCAGACCGCAGTATCCAT-3′	68
	mp65 R′	5′-GCTCCAGGTCTCGCTTCTT-3′	
16	mBcl-2 F′	5′-GTACCTGAACCGGCATCTG-3′	130
	mBcl-2 R′	5′-GCTGAGCAGGGTCTTCAGAG-3′	
17	mBax F′	5′-CTCCGTGAGCGGCTGCTTGTC-3′	82
	mBax R′	5′-GCCATGTGGGGGTCCCGAAG-3′	
18	mAtf3 F′	5′-GCTGGAGTCAGTTACCGTCAA-3′	93
	mAtf3 R′	5′-CGCCTCCTTTTCCTCTCAT-3′	
19	mCaspase 3 F′	5′-GAGGCTGACTTCCTGTATGCTT-3′	77
	mCaspase 3 R′	5′-AACCACGACCCGTCCTTT-3′	
20	mCaspase 7 F′	5′-CCGTCCACAATGACTGCTC-3′	78
	mCaspase 7 R′	5′-CCGAGTTGCTGTGGTCCT-3′	
21	mCaspase 8 F′	5′-TTGAACAATGAGATCCCCAAA-3′	70
	mCaspase 8 R′	5′-CCATTTCTACAAAAATTTCAAGCAG-3′	
22	mCaspase 9 F′	5′-TGCAGTCCCTCCTTCTCAG-3′	77
	mCaspase 9 R′	5′-GCTTTTTCCGGAGGAAGTTAAA-3′	
23	mIFNb1 F′	5′-CTGGCTTCCATCATGAACAA-3′	73
	mIFNb1 R′	5′-AGAGGGCTGTGGTGGAGAA-3′	
24	mIFNγ F′	5′-CACACCTGATTACTACCTTCT-3′	75
	mIFNγ R′	5′-CCTCAAACTTGGCAATACTC-3′	

## 5. Conclusions

Pardaxin is a peptide that effectively inhibited MN-11 cells in C57BL/6 mice. Nowadays, it is imperative to find novel anticancer drugs with increased selectivity. In the present study, we show that pardaxin induced apoptosis through an intrinsic apoptotic pathway in cancer cells, thus showing its potential to serve as an anticancer drug in *in vivo* experiments. To sum up, our study provides evidence from molecular and animal studies, which shows that pardaxin induces death of murine fibrosarcoma MN-11 cells. The data suggest that pardaxin retards tumor development through suppressing both cell proliferation and tumor vascularization. These findings suggest that pardaxin is a peptide that can be used as an effective drug to treat solid neoplasms. Further studies with a greater number of feline or dog patients are needed, also with a view of possible translation to therapies for human fibrosarcomas.

## Supplementary Files

Supplementary File 1: PDF-Document (PDF, 75 KB)
