# Supplementary material for: Pardaxin, a Fish Antimicrobial Peptide, Exhibits Antitumor Activity toward Murine Fibrosarcoma in Vitro and in Vivo
Source: Mar Drugs. 2012 Aug 22;10(8):1852–72. doi: 10.3390/md10081852 (PMC3447341; doi:10.3390/md10081852)

# Supplementary Information

**Figure S1.** Changes in glutamic oxaloacetic transaminase (GOT), glutamic pyruvic transaminase (GPT), total bilirubin (TBIL), albumin (ALB), total cholesterol (TCHO), triglyceride (TG), total protein (TP), uric acid, blood urea nitrogen (BUN), and creatine (Cre) after injection with different concentrations of pardaxin (low dose (L, 5 mg/kg; 0.1 mg/day), medium dose (M, 10 mg/kg; 0.2 mg/day), and high dose (H, 25 mg/kg; 0.5 mg/day), or untreated (untreat; D15-Ctrl-tumor only, D22-Ctrl-tumor only)) for 14 days. Serum was collected on days 15 and 22 after the primary injection. The control group was left untreated (wk0). wk1 indicates serum from mice bearing tumors after being injected with pardaxin for 7 days. Each bar represents the mean (from three determinations) and the standard error (SE). Data (mean ± SE) with different letters significantly differ ($p < 0.05$) among treatments.

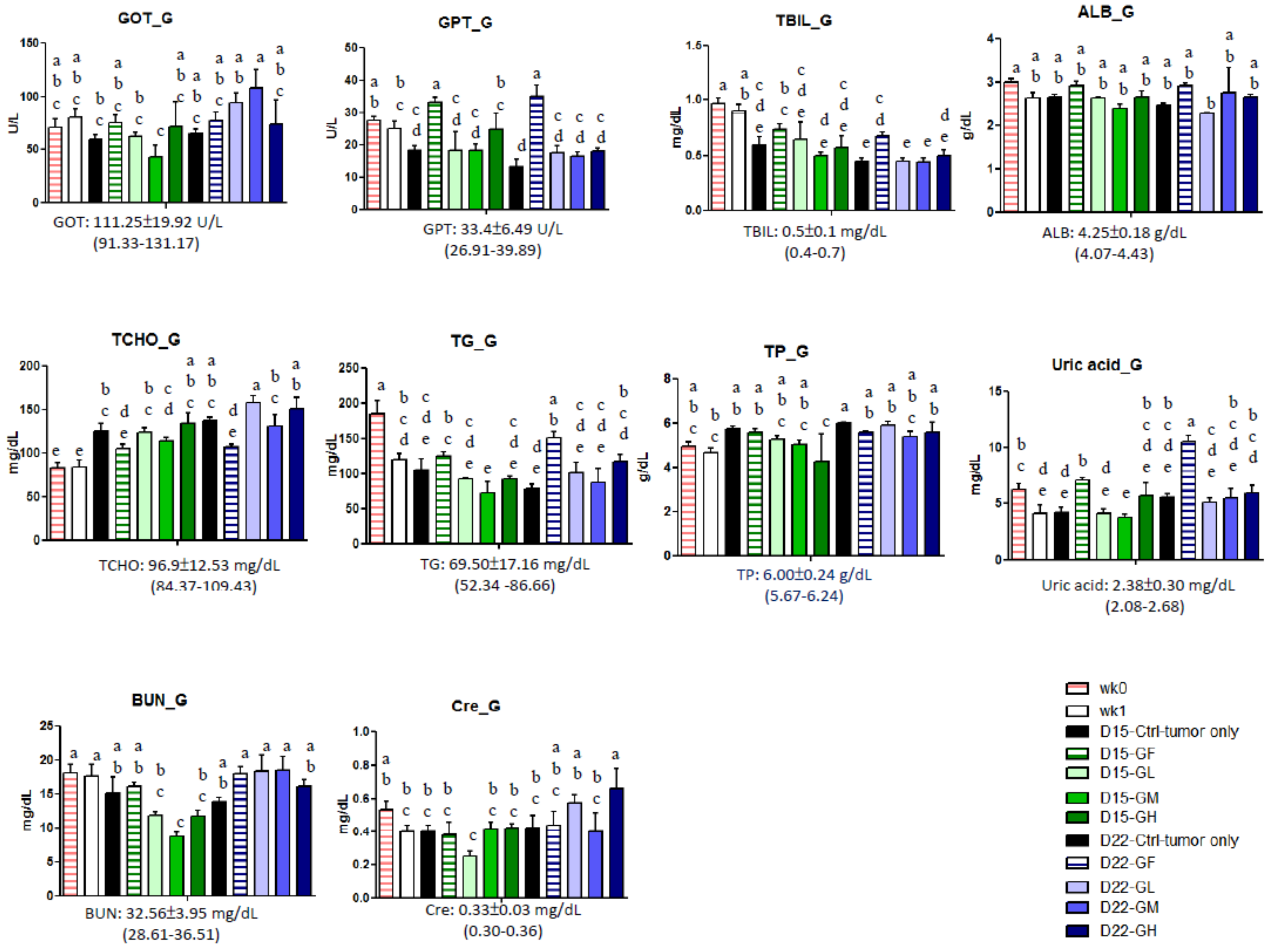

Supplement: Supplementary File 1: — PDF-Document (PDF, 75 KB) [file marinedrugs-10-01852-s001.pdf]
